# Mindfulness predicts less depression, anxiety, and social impairment in emergency care personnel: A longitudinal study

**DOI:** 10.1371/journal.pone.0260208

**Published:** 2021-12-15

**Authors:** Maren Westphal, Melanie Wall, Thomas Corbeil, Dagmar I. Keller, Monika Brodmann-Maeder, Ulrike Ehlert, Aristomenis Exadaktylos, Roland Bingisser, Birgit Kleim

**Affiliations:** 1 Department of Psychology, Pace University, Pleasantville, New York, United States of America; 2 Department of Psychiatry, Columbia University, New York, New York, United States of America; 3 Department of Biostatistics, Columbia University, New York, New York, United States of America; 4 Department of Emergency Medicine, University Hospital Zürich, Zürich, Switzerland; 5 Department of Emergency Medicine, Inselspital—University Hospital Bern, Bern, Switzerland; 6 Department of Psychology, Clinical Psychology and Psychotherapy, University of Zurich, Zurich, Switzerland; 7 Department of Emergency Medicine, University Hospital Basel, Basel, Switzerland; Chiba Daigaku, JAPAN

## Abstract

Medical personnel working in emergency rooms (ER) are at increased risk of mental health problems and suicidality. There is increasing evidence that mindfulness-based interventions can improve burnout and other mental health outcomes in health care providers. In contrast, few longitudinal prospective studies have examined protective functions of dispositional mindfulness in this population. The objective of this study was to examine whether mindfulness prospectively predicts anxiety, depression, and social impairment in a sample of emergency care professionals. The authors administered online surveys to ER personnel prior to work in ER, and at 3 and 6 months follow up. Participants were 190 ER personnel (73% residents, 16% medical students, 11% nurses). Linear mixed effects regression was used to model longitudinal 3-month and 6-month follow up of depression, anxiety, and social impairment. Predictors included time-varying contemporaneous work stressors, perceived social support at work and life events, and baseline dispositional mindfulness, demographics, and workplace characteristics. Mindfulness indexed when starting ER work predicted less depression, anxiety, and social impairment 6 months later. Mindfulness remained a strong predictor of mental health outcomes after controlling for time-varying stressful events in emergency care, negative life events, and social support at work. Mindfulness moderated the adverse impact of poor social support at work on depression. To our knowledge, this is the first longitudinal study to show that mindfulness prospectively and robustly predicts anxiety, depression, and social impairment. Results support the role of mindfulness as a potential resilience factor in at-risk health care providers.

## Introduction

Physicians and other medical personnel working in emergency rooms (ER) are routinely exposed to severe injuries and death and are at increased risk of developing mental health disorders and suicidality [1]. The scale and unpredictability of the Covid-19 pandemic has magnified the emotional toll of high-acuity care [2], along with hazardous working conditions and distressing triage decisions [3]. Mindfulness is a potential protective and modifiable variable [4] that is associated with better mental health in front-line workers such as firefighters [5,6] and emergency care professionals [7]. Mindfulness has been defined as an “awareness that emerges through paying attention on purpose, in the present moment, and nonjudgmentally, to the unfolding of experience moment by moment” [8] ^[p145]^. Mindfulness has been studied as a psychological trait that varies between and within individuals (i.e., dispositional mindfulness) and refers to the propensity or willingness to observe and sustain attention to present-moment experiences in day-to-day life [9,10]. Researchers have also operationalized mindfulness as a practice used to cultivate mindfulness (e.g., mindfulness meditation) and as a psychological state or process [11,12]. Studies have found that engaging in meditation and other mindfulness practices is associated with increased dispositional mindfulness over time [4], and both have been linked to better psychological health in diverse populations [4,9–14].

One of the ways that mindfulness is theorized to promote health is by shielding against the adverse impact of stress [15]. For example, a random population-based study found that dispositional mindfulness moderated the effects of perceived stress on depression [16]. Psychophysiological research has demonstrated that dispositional mindfulness modulates neuroendocrine and psychological stress responses to acute stress [17]. Experimental studies testing emotion regulatory functions of mindfulness have found that individuals high in dispositional mindfulness exhibit reduced reactivity to negative moods and emotional stimuli and situations [13,18–20].

Physicians are a population known to face high levels of stress, especially during training, and are at high risk for developing burnout [21], a condition typically studied in relation to work-related stress that is associated with both depression [22] and anxiety [23]. There is growing evidence from clinical trials conducted with physicians and other healthcare providers that interventions incorporating mindfulness practice can reduce burnout [24,25]. To this date, however, few longitudinal studies have examined whether dispositional mindfulness protects against stress in health care providers [26,27]. The present study examined the prospective relationship between dispositional mindfulness (i.e., mindfulness conceptualized as a psychological trait that varies between and within individuals) and symptoms of anxiety and depression as well as social impairment in a sample of ER personnel consisting mostly of residents.

As gatekeepers to care, ER personnel are frequently called upon to make split-second decisions based on limited information, which raises fear of making fatal errors. Intrusive memories and flashbacks of critical incidents are common in ER personnel and associated with greater depression, anxiety, and emotional exhaustion [28]. Compared to other medical specialties, physicians specializing in emergency medicine have higher rates of burnout [29]. For example, in a national study, 65% of ER physicians endorsed burnout, compared to 37% of all physicians and 28% in the general non-physician population [1]. More recently, a large prospective cohort study of medical students found that emergency medicine was one of four specialties that had the highest relative risk for burnout during residency [29].

Although most physicians do not develop symptoms severe enough to meet criteria for psychiatric disorders, several meta-analyses have shown that major depression and anxiety disorders tend to be substantially more prevalent among physicians-in-training compared to the general population. Between 20.9% and 43.2% of physicians-in-training screened positive for depression or depressive symptoms during residency [30] while medical students reported an overall prevalence of depression or depressive symptoms of 27.2%, and suicidal ideation of 11.1%, compared to a 7.2% prevalence of depression in the general population [31]. Similarly, a meta-analysis focused on trait anxiety found that medical students exhibited considerably higher anxiety compared to population norms [32]. A large cross-sectional study found that physicians-in-training were five times more likely to screen positive for major depressive disorder and eight times more likely to screen positive for generalized anxiety disorder compared to national estimates for an age matched comparison sample [33].

Social functioning has received relatively little attention in the literature on mental health in physicians, despite being an important aspect of quality of life and marker of emotional well-being [34,35]. For example, social functioning has been found to distinguish physicians with psychiatric and somatic problems from those without clinically significant levels of anxiety and depression [36]. Research also has revealed impaired social functioning in physicians seeking treatment for mental health problems [37], and a follow-up study showed that social functioning improved with treatment [38].

Given the relatively high prevalence of depression and anxiety in physicians in training reviewed above, and association between depression and greater rates of medical errors among physicians [39], identifying factors associated with resilience to stress in ER personnel and other at-risk health care providers is important for public health. A recent national survey study of pediatric residents found that higher dispositional mindfulness prospectively predicted lower perceived stress one year later [26]. After controlling for medical errors and other contextual risk factors, however, mindfulness no longer longitudinally predicted perceived stress [27]. This finding highlights the importance of measuring exposure to stressful events in testing protective benefits of mindfulness. To our knowledge, no published longitudinal study of mindfulness in health care professionals has controlled for both stressful events at work and negative life events.

Negative life events constitute a well-known risk factor in both clinical [40] and non-clinical populations [41]. Longitudinal research has shown that negative life events predict increased mental health problems among physicians-in-training [42] as well as established physicians [e.g., 43]. For example, a study conducted with medical students in the US found that the number of negative personal life events, such as personally experiencing a major illness strongly correlated with professional burnout and depression [44]. In a nationwide prospective cohort study of medical students [45], both negative life events and work-related stress were significant predictors of mental health problems severe enough to require treatment. In a large-scale Norwegian study, the majority of physicians endorsing suicidal thoughts attributed these thoughts predominantly to personal and family problems and to a lesser degree to work and social problems [46]. Therefore, in addition to measuring stressful events specific to the ER, we measured the number of negative life events participants reported in their personal lives over the course of the study.

Another factor that may influence mental health in physicians is social support at work [47]. Compared to senior physicians, physicians-in-training tend to have less power and autonomy at work and frequently receive insufficient support from experienced colleagues and supervisors [48]. In a previous cross-sectional study of ER personnel, we found that mindfulness was associated with less depression, anxiety, and burnout and moderated the impact of conflict with patients and personnel on anxiety [7]. Given prospective evidence that poor social support in the workplace predicts depression [49,50], we included social support at work as a predictor in our longitudinal investigation into protective functions of mindfulness.

In summary, the purpose of the present study was to examine prospective associations between mindfulness and three mental health outcomes (anxiety, depression, and social impairment) in ER personnel. Our study expanded on previous work by indexing mindfulness when individuals just started working in the ER. Controlling for relevant demographics, previous ER experience, work-related stress, negative life events, and social support at work, we hypothesized that greater mindfulness would prospectively predict reduced depression and anxiety, and better social functioning at 6 months in the job. We also investigated whether mindfulness moderates the effects of work-related stress, social support at work, and negative life events on depression and anxiety symptoms and social impairment.

## Materials and methods

### Participants and procedure

Participants were 190 emergency medicine (ER) staff employed in the Emergency Departments of three urban teaching hospitals in Switzerland (Zürich, Bern, and Basel). Only participants with complete baseline data and at least one follow-up were included in the analysis (*N* = 121). All participants were approached in person by the study team, provided written consent for their data to be used in research. The local Ethical Review Boards of all three Cantons (Zürich, Bern, and Basel) approved the study.

Data were then collected via anonymous questionnaires from the overall group of personnel starting at one time point (overall response rate: 57% across sites). The questionnaire comprised demographic and work-related questions as well as measures of anxiety and depressive symptoms, perceived social support at work, social adjustment, and mindfulness. The first wave of data collection occurred within two weeks after participants had started their work in the ER, the second after three months, and the third wave after six months.

### Measures

Demographic measures were assessed by self-report including sex, age, marital status, professional status (resident, nurse, or medical student) and level of experience in dealing with medical incidents in the ER, ranging from very unexperienced to very experienced.

Work-related stress was assessed with the *Stressful Events at Work Checklist* (SEW), a questionnaire that was specifically created for this study. The items for the SEW were derived from a previous study in which we had asked ER personnel to describe their most frequent intrusive memory of critical incidents encountered while working in the ER [28]. The memories were scored and categorized for content by independent raters with high interrater reliability (κ = 0.79). The SEW contains items that represent the six most frequently reported categories of critical incidents participants in this previous study had reported as having been associated with intrusive memories, which included: exposure to death, invasive treatment procedures, grieving family members, agitated family members, making a critical treatment error (self or colleague), and an “other” event option. For the present study, each event was rated for level of perceived stress, ranging from not stressful (1) to extremely stressful (4). A summary score was constructed across the six items by summing levels of perceived stress for each endorsed item.

Life events were measured with the *Life Events Questionnaire* (LEQ). This questionnaire measures the occurrence and impact of special life events within the last three months.

Items were selected from Norbeck [51], which compiles a variety of life events from different existing instruments, while format and instructions were derived from Sarason, Johnson and Siegel [52]. The selected 8 items covered life events in the following areas: health, work, living situation, intimate relationships, close friends and family, children, personal and social, and financial. For this study, we calculated the sum of all negative life events experienced over the course of the study (i.e., at Wave 1 and Wave 2).

Mindfulness was assessed with the German version of the dispositional *Mindful Attention Awareness Scale* (MAAS [53]. Based on a conceptualization of mindfulness as an attribute that varies between and within people [10], the scale measures the disposition to pay attention to and remain aware of and receptive to current experience. It contains 15 items that capture the experience of mindfulness and mindlessness in general terms (e.g., “I rush through activities without being really attentive to them”) as well as in specific day-to-day circumstances (e.g., “I find myself listening to someone with one ear, doing something else at the same time”; [10]. Respondents are asked how frequently they have the experience described in each statement using a 6-point Likert scale from 1 (*almost always*) to 6 (*almost never*). To score the MAAS, a mean of the 15 items is calculated, with higher scores reflecting higher levels of dispositional mindfulness. The scale’s reliability and validity has been established in both general (non-trainee) and mindfulness trainee populations, and intervention studies have demonstrated chances in its score over the course of mindfulness training [10,11]. The MAAS predicts self-regulated behavior and positive emotion in healthy people and has been associated with reduced stress and less mood disturbance in clinical populations [10]. The MAAS has demonstrated good internal consistency across a range of samples (α = .80–.87; [10]; present study α = .85) and strong test–retest reliability data over a 1-month time period (*r* = .81; [10]. The mean MAAS score in the present study was 4.29 (*SD* = 0.63), and the range for the MAAS score in our sample was 2.60 to 5.67.

Social support at work was measured with Ducharme and Martin’s ten-item scale which comprises five items each for affective (α = .85) and instrumental (α = .76) facets of coworker support [53]. Items are scored on a 3-point scale with higher scores reflecting higher levels of perceived affective and instrumental support, respectively. We combined the two subscales to create an index of overall social support at work by creating a mean of the 10 items. For this combined scale, the Cronbach alpha at baseline was α = 0.85.

Symptoms of anxiety and depression were assessed with the 14-item *Hospital Anxiety and Depression Scale* (HADS*)* [54]. Each item is scored from 0–3, resulting in scores between 0 and 21 for either anxiety or depression, with higher scores indicating greater symptomatology.

Social functioning was measured with the German version of the Social Adjustment Scale (SAS-SR) [55], which assesses functioning in social roles over the past two weeks across six domains [56]. The scale can be administered in full with 42 items rated on a 5-point scale or with focus on specific domains depending on study needs. For the purposes of this study, we administered only items that pertain to work and social functioning. Questions inquire about performance in social roles, quality of interpersonal relationships, friction with others, and feelings of satisfaction or dissatisfaction with social or leisure activities (e.g., interest, boredom, loneliness). The total SAS-SR score was calculated by averaging all items, with higher scores indicating higher impairment.

### Analysis

Descriptive frequencies of demographic and workplace characteristics were calculated. Means of the mindfulness variable were compared across levels of demographic and work characteristics and differences tested using one-way ANOVA with the exception of age which was tested using Pearson correlation. Linear mixed effects regression was used to model longitudinal 3-month and 6-month follow up of HADS and SAS-SR scores (3 separate models) with a random intercept to account for the within-subject correlation of repeated measures. Predictors included time-varying contemporaneous work stressors, social support at work and life events, and baseline mindfulness and demographic and workplace characteristics. Additional models incorporated interactions with mindfulness to investigate the possibility of effect modification by mindfulness on the relationship between work stressors, social supports, and life events on outcomes. When a significant interaction with mindfulness was identified, we summarized the relationship of predictor and outcome by high and low mindfulness values (i.e., one standard deviation above and below the mean mindfulness score). SAS 9.4 was used to conduct the analyses.

## Results

Table 1 summarizes demographic and workplace characteristics of the sample. Average age was 30.5 years. Most participants were female (63.64%), in a romantic relationship (71.07%), and worked as residents, i.e., started their advanced training in ER or related specialty that follows graduation (72.73%), and most were from the study site Bern {41.32%). Almost half (42.98%) of the ER personnel had little experience in emergency medicine at baseline. There were no significant associations between mindfulness and any of the demographic or workplace variables (all *p* values > 0.05).

**Table 1:** Sample demographics, work characteristics and associations with mindfulness (N = 121).

Variable	*n* (%) or Mean (SD)	Baseline Mindfulness Mean (SD)	*p* ^a^
		4.29 (0.63)	
**Age**	30.5 (4.96)		0.718
**Sex**			
Female	77 (63.64)	4.30 (0.61)	0.763
Male	44 (36.36)	4.27 (0.68)	
**Marital Status**			
Not in a Relationship	35 (28.93)	4.26 (0.59)	0.725
In a Relationship	86 (71.07)	4.30 (0.66)	
**Role**			
Residents	88 (72.73)	4.28 (0.66)	0.207
Nurses	14 (11.57)	4.55 (0.57)	
Medical Students	19 (15.70)	4.16 (0.51)	
**Previous ER experience**			
Very unexperienced	29 (23.97)	4.17 (0.64)	0.462
Little experience	52 (42.98)	4.34 (0.59)	
Experienced	37 (30.58)	4.28 (0.67)	
Very experienced	3 (2.48)	4.69 (0.80)	
**Site**			
Basel	29 (23.97)	4.17 (0.70)	0.500
Bern	50 (41.32)	4.32 (0.58)	
Zürich	42 (34.71)	4.33 (0.65)	

^a^*P*-values are from one-way ANOVAs of mindfulness differences across demographic and work characteristics; for age, the *p*-value is for the test of Pearson correlation with mindfulness.

Clinical symptoms of anxiety and depression were not above the clinical threshold in the overall group, although there was significant variance within our population, ranging from mild to moderate depression and anxiety symptoms, see Table 2.

**Table 2:** Mental health, social impairment, stressors and social support at baseline, 3 and 6 months.

	Baseline			3 Months			6 Months		
Variable	*N*	Mean	SD	*N*	Mean	SD	*N*	Mean	SD
Depression	121	2.45	2.46	91	2.76	2.99	105	2.43	2.66
Anxiety	121	5.11	2.97	91	4.41	3.60	105	4.20	3.07
Social Impairment	--	--	--	91	1.98	0.44	105	1.90	0.38
Work stress	--	--	--	91	6.47	3.85	106	7.03	4.16
Social support	--	--	--	90	2.58	0.30	105	2.54	0.34
Negative life events	121	1.14	1.52	91	1.46	1.83	106	1.12	1.41

Table 3 shows the results from the longitudinal models for the separate outcomes and all predictors. Mindfulness had significant protective effects on later depression (β = -0.26, *p* = 0.001), anxiety (β = -0.43, *p*<0.001) and social impairment (β = -0.16, *p*<0.021). After accounting for other factors, work stress was not associated with depression or anxiety, but significantly and independently predicted social impairment (β = 0.18, p = 0.006). On the other hand, negative life events were independently associated with higher anxiety, depression, and social impairment. Social support at work had a protective effect on depression and social functioning, but not on anxiety. Relationship status positively correlated with social functioning in that individuals in a relationship had less social impairment. Professional status correlated with anxiety in that residents were more anxious than nurses.

**Table 3:** Standardized regression coefficients for depression, anxiety, and social impairment predicted by mindfulness, work stress, social support at work, and negative life events (*N* = 121).

Predictor Variables	Depression		Anxiety		Social Impairment	
	β	*p*	β	*p*	β	*p*
Mindfulness	**-0.26**	**0.001**	**-0.43**	**<0.001**	**-0.16**	**0.021**
Work stress	0.06	0.360	0.08	0.173	**0.18**	**0.006**
Social support at work	**-0.17**	**0.008**	-0.01	0.910	**-0.24**	**<0.001**
Negative life events	**0.34**	**<0.001**	**0.23**	**<0.001**	**0.33**	**<0.001**
Sex (F vs. M)	-0.06	0.710	-0.06	0.718	-0.08	0.547
Age	0.10	0.176	-0.02	0.746	0.06	0.404
Relationship status (In a relationship vs. not in a relationship)	-0.15	0.363	-0.09	0.562	**-0.44**	**0.004**
Profession						** **
Medical students vs. nurses	-0.14	0.640	0.25	0.407	-0.12	0.666
Residents vs. nurses	0.32	0.174	**0.50**	**0.032**	0.32	0.139
Previous ER experience	-0.08	0.339	-0.07	0.376	-0.12	0.094

**Fig 1:**
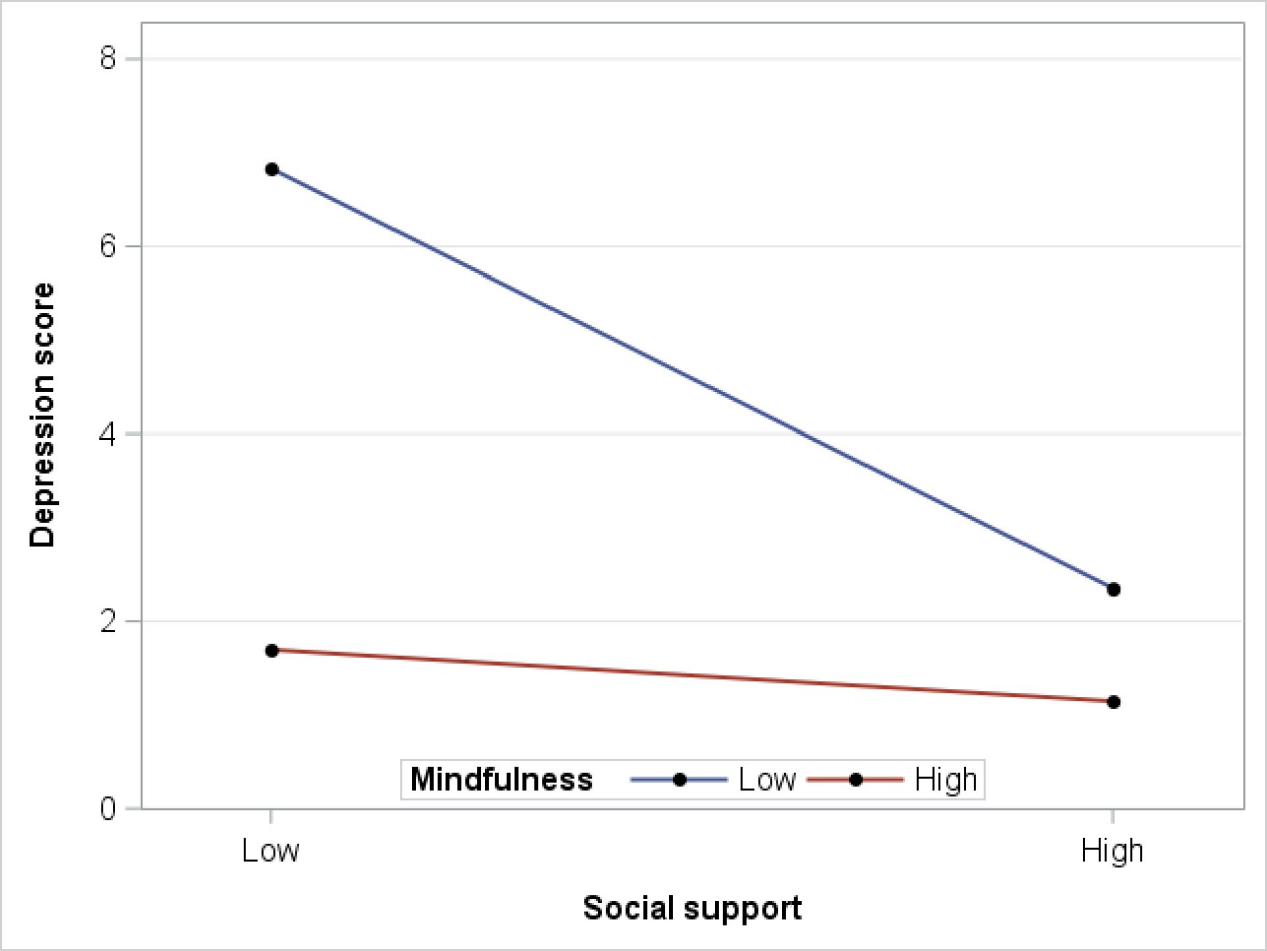
Mindfulness buffers the effect of low social support at work on depression (*N* = 121). Test of two-way interaction between mindfulness and social support on depression (t = 2.23, DF = 113, p = 0.027; low mindfulness β = -2.80, t = -3.45, p<0.001; high mindfulness β = -0.3447, t = -0.50, p = 0.618).

A significant interaction emerged between mindfulness and one of the significant primary predictors. Mindfulness moderated the association between social support at work and depression. Specifically, while high social support was associated with less depression, this association was significant only among participants with low mindfulness (Beta = -2.80, p <0.001), but not among those with high mindfulness, see Fig 1. No other significant interactions were detected between mindfulness and our primary predictors for any of the three outcomes.

## Discussion

Few studies have examined if mindfulness prospectively predicts better mental health in at risk populations. Knowledge of protective benefits of mindfulness can help support stress-exposed individuals early on, which may prevent negative mental health outcomes. The current study documented protective benefits of mindfulness indexed before starting work in an ER department. Using a longitudinal design with three waves of data collection, we found that mindfulness predicted lower levels of depression, anxiety, and social impairment after controlling for work-related stress, negative life events, and perceived social support at work. Effects were strongest for anxiety, followed by depression, and social impairment. Further, low mindfulness was uniquely associated with increased symptoms of depression in the presence of poor social support at work.

This robust prospective relationship between mindfulness and anxiety is consistent with meta-analytic evidence that mindfulness-based interventions are effective in reducing anxiety in physicians and medical students [57]. Our findings extend this literature by demonstrating that mindfulness prospectively predicts less anxiety in ER personnel, a population facing high levels of stress.

We also found a significant prospective relationship between mindfulness and better social functioning. There is increasing evidence linking mindfulness to better emotion regulation ability [13,58–61], which in turn is associated with less anxiety and depression [62] as well as better social functioning [63]. Experimental research has demonstrated a positive relationship between mindfulness and constructive responses to interpersonal conflict [64], suggesting that mindfulness may help manage difficult social interactions.

In addition to demonstrating that dispositional mindfulness robustly predicts better mental health, our findings highlight the importance of contextual predictors of emotional well-being in health care providers. Specifically, the finding that negative life events were robustly associated with all three mental health outcomes is consistent with large-scale longitudinal studies that identified negative life events as a risk factor for mental health problems in physicians and physicians-in-training [42,44].The only significant demographic predictors were relationship status and profession. Given that decreased social participation and lack of close social relationships are core features of social impairment [65], one might expect that those in a relationship would report better social functioning than those without. Less predictable was the finding that residents had higher levels of anxiety than nurses. While the small number of nurses in our sample limits comparisons, it is possible that specific challenges associated with residency may have contributed to this group difference. Residency typically coincides with the family-building stage of young physicians, which increases the possibility of work-family conflict [43,66]. Future studies including balanced samples of nurses and physicians would be needed to examine profession-specific differences in the stress-anxiety relationship. On a methodological level, the finding that social impairment was associated with a greater number of predictors than anxiety and depression suggests that including measures of social functioning in future studies may help capture more fully the adverse effects of psychosocial stressors on health care providers.

The finding that low mindfulness was associated with elevated levels of depression in individuals with poor social support at work is noteworthy and consistent with extensive evidence for a strong relationship between social support and depression [67]. The moderating effect of mindfulness in the context of an unsupportive work environment also converges with findings that dispositional mindfulness protects against distress arising from rejection, a highly aversive experience that signals the threat of social isolation [68]. Mindfulness in interpersonal communication may help detect and use social cues in a goal-directed manner [69] while mindlessness increases susceptibility to cognitive biases [70]. Extensive evidence that depression is associated with overactivation of negative cognitive biases [71] provides support for the idea that mindfulness may protect against depression at least in part through increasing flexibility in processing and regulating emotions in social relationships [19].

Given that depression has been linked to greater rates of medical errors among physicians [39], our findings have implications for patient care. As a malleable protective factor, dispositional mindfulness may help to understand differences in the well-being of health care professionals, index changes in mindfulness during mindfulness-based interventions and examine potential associations with improvements in patient care. For example, higher dispositional mindfulness has been associated with less depression and lower stress in medical students [72], improved self-care among physicians-in-training [73], and more patient-centered communication and higher patient satisfaction among physicians [74]. Research suggests that stress-reduction interventions for health care providers that incorporate mindfulness training can increase empathy [75], and positively affect treatment course and clinical outcomes [76].

Our study is not without limitations. We investigated ER personnel and this may constitute a selective (and potentially already resilient) group of individuals opting into this profession. Our results may thus not be directly transferable to other at-risk populations. Moreover, we investigated mindfulness as a trait prior to starting work at the ER. To examine the relation between mindfulness and resilience in future studies, it would be important to measure mindfulness at multiple time points over the course of working in the ER. To examine the relation between mindfulness and resilience in future studies, it would be important to measure mindfulness at multiple time points over the course of working in acute care settings.

Overall, the results of the current prospective study underscore the importance of mindfulness as a protective factor in emergency care and other high stress occupational environments. The majority of ER personnel in the current study were completing their residency, which is considered to be the most demanding and exhausting stage of medical training over the course of a physician’s career, in part owing to the combination of low job control and high work demands [26,77]. Our findings suggest that mindfulness interventions for physicians and other health care professionals may be beneficial at both individual and institutional levels by improving mental health and facilitating supportive relationships at work.

## References

[1] Shanafelt TD, Boone S, Tan L, et al. Burnout and satisfaction with work-life balance among us physicians relative to the general us population. Archives of Internal Medicine. 2012;172(18):1377–85. doi: 10.1001/archinternmed.2012.3199 22911330

[2] Greenberg N, Weston D, Hall C, Caulfield T, Williamson V, Fong K. Mental health of staff working in intensive care during COVID-19. Occupational Medicine. 2021. doi: 10.1093/occmed/kqaa220 33434920 PMC7928568

[3] Shaukat N, Ali DM, Razzak J. Physical and mental health impacts of COVID-19 on healthcare workers: a scoping review. International Journal of Emergency Medicine. 2020;13(1):40. doi: 10.1186/s12245-020-00299-5 32689925 PMC7370263

[4] Goldberg SB, Tucker RP, Greene PA, Simpson TL, Hoyt WT, Kearney DJ, et al. What Can We Learn from Randomized Clinical Trials About the Construct Validity of Self-Report Measures of Mindfulness? A Meta-Analysis. Mindfulness. 2018. doi: 10.1007/s12671-018-1032-y 31263511 PMC6602541

[5] Smith BW, Ortiz JA, Steffen LE, Tooley EM, Wiggins KT, Yeater EA, et al. Mindfulness is associated with fewer PTSD symptoms, depressive symptoms, physical symptoms, and alcohol problems in urban firefighters. Journal of Consulting and Clinical Psychology. 2011;79(5):613. doi: 10.1037/a0025189 21875175

[6] Counson I, Hosemans D, Lal TJ, Mott B, Harvey SB, Joyce S. Mental health and mindfulness amongst Australian fire fighters. BMC Psychol. 2019;7(1):34. doi: 10.1186/s40359-019-0311-2 31200776 PMC6570940

[7] Westphal M, Bingisser M-B, Feng T, Wall M, Blakley E, Bingisser R, et al. Protective benefits of mindfulness in emergency room personnel. Journal of affective disorders. 2015;175:79–85. doi: 10.1016/j.jad.2014.12.038 25597793

[8] Kabat-Zinn J. Mindfulness-based interventions in context: Past, present, and future. Clinical Psychology: Science and Practice. 2003;10(2):144–56.

[9] Baer RA, Smith GT, Hopkins J, Krietemeyer J, Toney L. Using Self-Report Assessment Methods to Explore Facets of Mindfulness. Assessment. 2006;13(1):27–45. doi: 10.1177/1073191105283504 16443717

[10] Brown KW, Ryan RM. The benefits of being present: Mindfulness and its role in psychological well-being. Journal of Personality and Social Psychology. 2003;84(4):822–48. doi: 10.1037/0022-3514.84.4.822 12703651

[11] Brown KW, Ryan RM, Creswell JD. Mindfulness: Theoretical Foundations and Evidence for its Salutary Effects. Psychological Inquiry. 2007;18(4):211–37.

[12] Lau MA, Bishop SR, Segal ZV, Buis T, Anderson ND, Carlson L, et al. The toronto mindfulness scale: Development and validation. Journal of Clinical Psychology. 2006;62(12):1445–67. doi: 10.1002/jclp.20326 17019673

[13] Guendelman S, Medeiros S, Rampes H. Mindfulness and Emotion Regulation: Insights from Neurobiological, Psychological, and Clinical Studies. Frontiers in Psychology. 2017;8(220). 10.3389/fpsyg.2017.00220 PMC5337506 28321194

[14] Keng S-L, Smoski MJ, Robins CJ. Effects of mindfulness on psychological health: a review of empirical studies. Clin Psychol Rev. 2011;31(6):1041–56. doi: 10.1016/j.cpr.2011.04.006 21802619 PMC3679190

[15] Creswell JD, Lindsay EK. How Does Mindfulness Training Affect Health? A Mindfulness Stress Buffering Account. Current Directions in Psychological Science. 2014;23(6):401–7.

[16] Bränström R, Duncan LG, Moskowitz JT. The association between dispositional mindfulness, psychological well‐being, and perceived health in a Swedish population‐based sample. British journal of health psychology. 2011;16(2):300–16. 21489058 10.1348/135910710X501683 PMC3762484

[17] Brown KW, Weinstein N, Creswell JD. Trait mindfulness modulates neuroendocrine and affective responses to social evaluative threat. Psychoneuroendocrinology. 2012;37(12):2037–41. doi: 10.1016/j.psyneuen.2012.04.003 22626868 PMC5087919

[18] Arch JJ, Craske MG. Mechanisms of mindfulness: Emotion regulation following a focused breathing induction. Behaviour Research and Therapy. 2006;44(12):1849–58. doi: 10.1016/j.brat.2005.12.007 16460668

[19] Britton WB, Shahar B, Szepsenwol O, Jacobs WJ. Mindfulness-Based Cognitive Therapy Improves Emotional Reactivity to Social Stress: Results from a Randomized Controlled Trial. Behavior Therapy. 2012;43(2):365–80. doi: 10.1016/j.beth.2011.08.006 22440072 PMC3495556

[20] Raes F, Dewulf D, Van Heeringen C, Williams JMG. Mindfulness and reduced cognitive reactivity to sad mood: Evidence from a correlational study and a non-randomized waiting list controlled study. Behaviour Research and Therapy. 2009;47(7):623–7. doi: 10.1016/j.brat.2009.03.007 19362705

[21] Zhou AY, Panagioti M, Esmail A, Agius R, Van Tongeren M, Bower P. Factors Associated With Burnout and Stress in Trainee Physicians: A Systematic Review and Meta-analysis. JAMA Network Open. 2020;3(8):e2013761–e. doi: 10.1001/jamanetworkopen.2020.13761 32809031 PMC7435345

[22] Orosz A, Federspiel A, Haisch S, Seeher C, Dierks T, Cattapan K. A biological perspective on differences and similarities between burnout and depression. Neuroscience & Biobehavioral Reviews. 2017;73:112–22. doi: 10.1016/j.neubiorev.2016.12.005 27993607

[23] Koutsimani P, Montgomery A, Georganta K. The Relationship Between Burnout, Depression, and Anxiety: A Systematic Review and Meta-Analysis. Frontiers in Psychology. 2019;10(284). 10.3389/fpsyg.2019.00284 PMC6424886 30918490

[24] West CP, Dyrbye LN, Erwin PJ, Shanafelt TD. Interventions to prevent and reduce physician burnout: a systematic review and meta-analysis. The Lancet. 2016;388(10057):2272–81. doi: 10.1016/S0140-6736(16)31279-X 27692469

[25] Kriakous SA, Elliott KA, Lamers C, Owen R. The Effectiveness of Mindfulness-Based Stress Reduction on the Psychological Functioning of Healthcare Professionals: a Systematic Review. Mindfulness. 2021;12(1):1–28. 10.1007/s12671-020-01500-9 PMC7511255 32989406

[26] Kemper KJ, McClafferty H, Wilson PM, Serwint JR, Batra M, Mahan JD, et al. Do Mindfulness and Self-Compassion Predict Burnout in Pediatric Residents? Academic Medicine. 2019;94(6). doi: 10.1097/ACM.0000000000002546 30520809

[27] Kemper KJ, Schwartz A, Wilson PM, Mahan JD, Schubert CJ, Staples BB, et al. Burnout in Pediatric Residents: Three Years of National Survey Data. Pediatrics. 2020;145(1):11. doi: 10.1542/peds.2019-1030 31843859

[28] Kleim B, Bingisser M-B, Westphal M, Bingisser R. Frozen moments: flashback memories of critical incidents in emergency personnel. Brain and Behavior. 2015;5(7):e00325. doi: 10.1002/brb3.325 26221567 PMC4511283

[29] Dyrbye LN, Burke SE, Hardeman RR, et al. Association of clinical specialty with symptoms of burnout and career choice regret among us resident physicians. JAMA. 2018;320(11):1114–30. doi: 10.1001/jama.2018.12615 30422299 PMC6233627

[30] Mata DA, Ramos MA, Bansal N, et al. Prevalence of depression and depressive symptoms among resident physicians: A systematic review and meta-analysis. JAMA. 2015;314(22):2373–83. doi: 10.1001/jama.2015.15845 26647259 PMC4866499

[31] Rotenstein LS, Ramos MA, Torre M, et al. Prevalence of depression, depressive symptoms, and suicidal ideation among medical students: A systematic review and meta-analysis. JAMA. 2016;316(21):2214–36. doi: 10.1001/jama.2016.17324 27923088 PMC5613659

[32] Dyrbye LN, Thomas MR, Shanafelt TD. Systematic review of depression, anxiety, and other indicators of psychological distress among US and Canadian medical students. Academic Medicine. 2006;81(4):354–73. doi: 10.1097/00001888-200604000-00009 16565188

[33] Mousa OY, Dhamoon MS, Lander S, Dhamoon AS. The MD blues: under-recognized depression and anxiety in medical trainees. PloS one. 2016;11(6):e0156554. doi: 10.1371/journal.pone.0156554 27286249 PMC4902257

[34] Bech P. Social functioning: should it become an endpoint in trials of antidepressants? CNS drugs. 2005;19(4):313–24. doi: 10.2165/00023210-200519040-00004 15813645

[35] Murphy H, Murphy EK. Comparing quality of life using the World Health Organization Quality of Life measure (WHOQOL-100) in a clinical and non-clinical sample: Exploring the role of self-esteem, self-efficacy and social functioning. Journal of Mental Health. 2006;15(3):289–300.

[36] Stavem K, Hofoss D, Aasland OG. Work characteristics and morbidity as predictors of self-perceived health status in Norwegian physicians. Scandinavian Journal of Social Medicine. 2003;31(5):375–81. doi: 10.1080/14034940210165127 14555374

[37] Brooks SK, Chalder T, Gerada C. Doctors vulnerable to psychological distress and addictions: Treatment from the Practitioner Health Programme. Journal of Mental Health. 2011;20(2):157–64. doi: 10.3109/09638237.2011.556168 21542716

[38] Brooks SK, Gerada C, Chalder T. Doctors and dentists with mental ill health and addictions: Outcomes of treatment from the Practitioner Health Programme. Journal of Mental Health. 2013;22(3):237–45. doi: 10.3109/09638237.2012.734647 23323773

[39] Pereira-Lima K, Mata DA, Loureiro SR, Crippa JA, Bolsoni LM, Sen S. Association Between Physician Depressive Symptoms and Medical Errors A Systematic Review and Meta-analysis. Jama Network Open. 2019;2(11):14. doi: 10.1001/jamanetworkopen.2019.16097 31774520 PMC6902829

[40] Cronkite RC, Woodhead EL, Finlay A, Timko C, Unger Hu K, Moos RH. Life stressors and resources and the 23-year course of depression. Journal of Affective Disorders. 2013;150(2):370–7. doi: 10.1016/j.jad.2013.04.022 23688913

[41] Asselmann E, Wittchen H-U, Lieb R, Höfler M, Beesdo-Baum K. Danger and loss events and the incidence of anxiety and depressive disorders: a prospective-longitudinal community study of adolescents and young adults. Psychological medicine. 2015;45(1):153–63. doi: 10.1017/S0033291714001160 25065411

[42] Midtgaard M, Ekeberg Ø, Vaglum P, Tyssen R. Mental health treatment needs for medical students: a national longitudinal study. European Psychiatry. 2008;23(7):505–11. doi: 10.1016/j.eurpsy.2008.04.006 18774273

[43] Hertzberg TK, Rø KI, Vaglum PJW, Moum T, Røvik JO, Gude T, et al. Work-home interface stress: an important predictor of emotional exhaustion 15 years into a medical career. Industrial health. 2016;54(2):139–48. doi: 10.2486/indhealth.2015-0134 26538002 PMC4821897

[44] Dyrbye LN, Thomas MR, Huntington JL, Lawson KL, Novotny PJ, Sloan JA, et al. Personal life events and medical student burnout: a multicenter study. Academic Medicine. 2006;81(4):374–84. doi: 10.1097/00001888-200604000-00010 16565189

[45] Tyssen R, Vaglum P, Grønvold NT, Ekeberg Ø. The impact of job stress and working conditions on mental health problems among junior house officers. A nationwide Norwegian prospective cohort study. Medical Education. 2000;34(5):374–84. doi: 10.1046/j.1365-2923.2000.00540.x 10760123

[46] Hem E, Grønvold NT, Aasland OG, Ekeberg Ø. The prevalence of suicidal ideation and suicidal attempts among Norwegian physicians. Results from a cross-sectional survey of a nationwide sample. European Psychiatry. 2000;15(3):183–9. doi: 10.1016/s0924-9338(00)00227-3 10881215

[47] Bernburg M, Vitzthum K, Groneberg DA, Mache S. Physicians9 occupational stress, depressive symptoms and work ability in relation to their working environment: a cross-sectional study of differences among medical residents with various specialties working in German hospitals. BMJ open. 2016;6(6):e011369. doi: 10.1136/bmjopen-2016-011369 27311909 PMC4916614

[48] Fischer N, Degen C, Li J, Loerbroks A, Müller A, Angerer P. Associations of psychosocial working conditions and working time characteristics with somatic complaints in German resident physicians. International Archives of Occupational and Environmental Health. 2016;89(4):583–92. doi: 10.1007/s00420-015-1096-0 26542379

[49] Rugulies R, Bültmann U, Aust B, Burr H. Psychosocial Work Environment and Incidence of Severe Depressive Symptoms: Prospective Findings from a 5-Year Follow-up of the Danish Work Environment Cohort Study. American Journal of Epidemiology. 2006;163(10):877–87. doi: 10.1093/aje/kwj119 16571741

[50] Stoetzer U, Ahlberg G, Johansson G, Bergman P, Hallsten L, Forsell Y, et al. Problematic Interpersonal Relationships at Work and Depression: A Swedish Prospective Cohort Study. Journal of Occupational Health. 2009;51(2):144–51. doi: 10.1539/joh.l8134 19225218

[51] Norbeck JS. Modification of life event questionnaires for use with female respondents. Research in Nursing & Health. 1984;7(1):61–71. doi: 10.1002/nur.4770070110 6565302

[52] Sarason IG, Johnson JH, Siegel JM. Assessing the impact of life changes: Development of the Life Experiences Survey. Journal of Consulting and Clinical Psychology. 1978;46(5):932–46. doi: 10.1037//0022-006x.46.5.932 701572

[53] Ducharme LJ, Martin JK. Unrewarding work, coworker support, and job satisfaction: A test of the buffering hypothesis. Work and occupations. 2000;27(2):223–43.

[54] Zigmond AS, Snaith RP. The hospital anxiety and depression scale. Acta psychiatrica scandinavica. 1983;67(6):361–70. doi: 10.1111/j.1600-0447.1983.tb09716.x 6880820

[55] Geue K, Richter R, Buttstädt M, Brähler E, Singer S. Der Fragebogen zur sozialen Integration (FSI)-Psychometrische Testung und Akzeptanz bei Patienten mit hämatologischen Neoplasien. Zeitschrift für Psychosomatische Medizin und Psychotherapie. 2014;60(1):3–16. 24615234

[56] Gameroff MJ, Wickramaratne P, Weissman MM. Testing the Short and Screener versions of the Social Adjustment Scale—Self-report (SAS-SR). International Journal of Methods in Psychiatric Research. 2012;21(1):52–65. doi: 10.1002/mpr.358 22139969 PMC3433762

[57] Regehr C, Glancy D, Pitts A, LeBlanc VR. Interventions to Reduce the Consequences of Stress in Physicians: A Review and Meta-Analysis. The Journal of Nervous and Mental Disease. 2014;202(5):353–9. doi: 10.1097/NMD.0000000000000130 24727721

[58] Chiesa A, Serretti A, Jakobsen JC. Mindfulness: Top–down or bottom–up emotion regulation strategy? Clin Psychol Rev. 2013;33(1):82–96. doi: 10.1016/j.cpr.2012.10.006 23142788

[59] Bullis JR, Bøe HJ, Asnaani A, Hofmann SG. The benefits of being mindful: Trait mindfulness predicts less stress reactivity to suppression. Journal of Behavior Therapy and Experimental Psychiatry. 2014;45(1):57–66. doi: 10.1016/j.jbtep.2013.07.006 23994223

[60] Roemer L, Williston SK, Rollins LG. Mindfulness and emotion regulation. Current Opinion in Psychology. 2015;3:52–7.

[61] Arch JJ, Craske MG. Laboratory stressors in clinically anxious and non-anxious individuals: The moderating role of mindfulness. Behaviour Research and Therapy. 2010;48(6):495–505. doi: 10.1016/j.brat.2010.02.005 20303471

[62] Aldao A, Nolen-Hoeksema S, Schweizer S. Emotion-regulation strategies across psychopathology: A meta-analytic review. Clin Psychol Rev. 2010;30(2):217–37. doi: 10.1016/j.cpr.2009.11.004 20015584

[63] English T, John OP, Srivastava S, Gross JJ. Emotion regulation and peer-rated social functioning: A 4-year longitudinal study. Journal of Research in Personality. 2012;46(6):780–4. doi: 10.1016/j.jrp.2012.09.006 23471162 PMC3587109

[64] Barnes S, Brown KW, Krusemark E, Campbell WK, Rogge RD. The role of mindfulness in romantic relationship satisfaction and responses to relationship stress. Journal of Marital and Family Therapy. 2007;33(4):482–500. doi: 10.1111/j.1752-0606.2007.00033.x 17935531

[65] Goldman HH, Skodol AE, Lave TR. Revising Axis V for "DSM-IV": A Review of Measures of Social Functioning. American Journal of Psychiatry. 1992;149(9):1148. doi: 10.1176/ajp.149.9.1148 1386964

[66] Fuß I, Nübling M, Hasselhorn H-M, Schwappach D, Rieger MA. Working conditions and Work-Family Conflict in German hospital physicians: psychosocial and organisational predictors and consequences. BMC Public Health. 2008;8(1):353. doi: 10.1186/1471-2458-8-353 18840296 PMC2577658

[67] Santini ZI, Koyanagi A, Tyrovolas S, Mason C, Haro JM. The association between social relationships and depression: A systematic review. Journal of Affective Disorders. 2015;175:53–65. doi: 10.1016/j.jad.2014.12.049 25594512

[68] Maymin PZ, Langer EJ. Cognitive biases and mindfulness. Humanities and Social Sciences Communications. 2021;8(1):40.

[69] Burgoon JK, Berger CR, Waldron VR. Mindfulness and interpersonal communication. Journal of Social Issues. 2000;56(1):105–27.

[70] Martelli AM, Chester DS, Warren Brown K, Eisenberger NI, DeWall CN. When less is more: mindfulness predicts adaptive affective responding to rejection via reduced prefrontal recruitment. Social Cognitive and Affective Neuroscience. 2018;13(6):648–55. doi: 10.1093/scan/nsy037 29868921 PMC6022565

[71] LeMoult J, Gotlib IH. Depression: A cognitive perspective. Clin Psychol Rev. 2019;69:51–66. doi: 10.1016/j.cpr.2018.06.008 29961601 PMC11884012

[72] Alzahrani AM, Hakami A, AlHadi A, Batais MA, Alrasheed AA, Almigbal TH. The interplay between mindfulness, depression, stress and academic performance in medical students: A Saudi perspective. PLOS ONE. 2020;15(4):e0231088. doi: 10.1371/journal.pone.0231088 32243468 PMC7122761

[73] Slonim J, Kienhuis M, Di Benedetto M, Reece J. The relationships among self-care, dispositional mindfulness, and psychological distress in medical students. Med Educ Online. 2015;20:27924–. doi: 10.3402/meo.v20.27924 26112354 PMC4481049

[74] Beach MC, Roter D, Korthuis PT, Epstein RM, Sharp V, Ratanawongsa N, et al. A Multicenter Study of Physician Mindfulness and Health Care Quality. Annals of Family Medicine. 2013;11(5):421–8. doi: 10.1370/afm.1507 24019273 PMC3767710

[75] Krasner MS, Epstein RM, Beckman H, Suchman AL, Chapman B, Mooney CJ, et al. Association of an educational program in mindful communication with burnout, empathy, and attitudes among primary care physicians. Jama. 2009;302(12):1284–93. doi: 10.1001/jama.2009.1384 19773563

[76] Grepmair L, Mitterlehner F, Loew T, Bachler E, Rother W, Nickel M. Promoting Mindfulness in Psychotherapists in Training Influences the Treatment Results of Their Patients: A Randomized, Double-Blind, Controlled Study. Psychotherapy and Psychosomatics. 2007;76(6):332–8. doi: 10.1159/000107560 17917468

[77] Thomas NK. Resident Burnout. JAMA. 2004;292(23):2880–9. doi: 10.1001/jama.292.23.2880 15598920

